# Lectures on Bright’s Disease

**Published:** 1889-08

**Authors:** 


**Lectures on Bright's Disease.** By ROBERT SAUNDBY, M. D., Edin. Fellow of the Royal College of Physicians, London, Emeritus Senior President of the Royal Medical Society; Fellow of the Royal Medico-Chirurgical Society, etc., etc.; with fifty illustrations. Octavo, cloth, pp. vi., 290. New York: E. B. Treat. 1889. Price $2.75.

We have recently given space to the consideration of the subject of the Diseases of the Kidneys, and therefore an extended notice of this work will not be required. It is sufficient to say that this volume of Dr. Saundby's is a valuable contribution. He has a clear grasp of the facts that have passed before him during many years, and presents his conclusions in a concise and satisfactory manner. The whole subject of Bright's Disease receives attention; the present state of our knowledge is finely stated, and the author's own experience carefully detailed. The work will be found to repay diligent study. The illustrations are very good; a well-selected Bibliography follows each chapter, and a complete alphabetical index closes the volume. It appears as the sixteenth volume of the series of Treat's Medical Classics, and will, no doubt, be received with great favor by all students.

